# Estimating Patient-Specific Relative Benefit of Adding Biologics to Conventional Rheumatoid Arthritis Treatment: An Individual Participant Data Meta-Analysis

**DOI:** 10.1001/jamanetworkopen.2023.21398

**Published:** 2023-06-30

**Authors:** Yan Luo, Konstantina Chalkou, Satoshi Funada, Georgia Salanti, Toshi A. Furukawa

**Affiliations:** 1 Department of Health Promotion and Human Behavior, School of Public Health, Graduate School of Medicine, Kyoto University, Kyoto, Japan; 2 Population Health and Policy Research Unit, Medical Education Center, Graduate School of Medicine, Kyoto University, Kyoto, Japan; 3 Institute of Social and Preventive Medicine, University of Bern, Bern, Switzerland; 4 Graduate School for Health Sciences, University of Bern, Bern, Switzerland; 5 Department of Preventive Medicine and Public Health, School of Medicine, Keio University, Tokyo, Japan

## Abstract

**Importance:**

Current evidence remains ambiguous regarding whether biologics should be added to conventional treatment of rheumatoid arthritis for specific patients, which may cause potential overuse or treatment delay.

**Objectives:**

To estimate the benefit of adding biologics to conventional antirheumatic drugs for the treatment of rheumatoid arthritis given baseline characteristics.

**Data Sources:**

Cochrane CENTRAL, Scopus, MEDLINE, and the World Health Organization International Clinical Trials Registry Platform were searched for articles published from database inception to March 2, 2022.

**Study Selection:**

Randomized clinical trials comparing certolizumab plus conventional antirheumatic drugs with placebo plus conventional drugs were selected.

**Data Extraction and Synthesis:**

Individual participant data of the prespecified outcomes and covariates were acquired from the Vivli database. A 2-stage model was fitted to estimate patient-specific relative outcomes of adding certolizumab vs conventional drugs only. Stage 1 was a penalized logistic regression model to estimate the baseline expected probability of the outcome regardless of treatment using baseline characteristics. Stage 2 was a bayesian individual participant data meta-regression model to estimate the relative outcomes for a particular baseline expected probability. Patient-specific results were displayed interactively on an application based on a 2-stage model.

**Main Outcomes and Measures:**

The primary outcome was low disease activity or remission at 3 months, defined by 3 disease activity indexes (ie, Disease Activity Score based on the evaluation of 28 joints, Clinical Disease Activity Index, or Simplified Disease Activity Index).

**Results:**

Individual participant data were obtained from 3790 patients (2996 female [79.1%] and 794 male [20.9%]; mean [SD] age, 52.7 [12.3] years) from 5 large randomized clinical trials for moderate to high activity rheumatoid arthritis with usable data for 22 prespecified baseline covariates. Overall, adding certolizumab was associated with a higher probability of reaching low disease activity. The odds ratio for patients with an average baseline expected probability of the outcome was 6.31 (95% credible interval, 2.22-15.25). However, the benefits differed in patients with different baseline characteristics. For example, the estimated risk difference was smaller than 10% for patients with either low or high baseline expected probability.

**Conclusions and Relevance:**

In this individual participant data meta-analysis, adding certolizumab was associated with more effectiveness for rheumatoid arthritis in general. However, the benefit was uncertain for patients with low or high baseline expected probability, for whom other evaluations were necessary. The interactive application displaying individual estimates may help with treatment selection.

## Key Points

**Question:**

Should biologics be added to conventional treatment for specific patients with rheumatoid arthritis considering the short-term benefit?

**Findings:**

In this meta-analysis of individual participant data from 3790 patients, the addition of certolizumab, a tumor necrosis factor α inhibitor, to conventional rheumatoid arthritis treatment was associated with an increased probability of reaching low disease activity in general. However, an interactive application based on a 2-stage model, which can visualize the estimated results for individual patients, showed that the absolute benefits differed in patients with different baseline characteristics.

**Meaning:**

These findings suggest that the benefit of adding biologics to conventional rheumatoid arthritis treatment is uncertain for specific patients, and the interactive application based on a 2-stage model may help with treatment selection in practice.

## Teaser

This individual participant data meta-analysis estimates patient-specific outcomes of adding biologics to conventional synthetic disease-modifying antirheumatic drugs for the treatment of rheumatoid arthritis.

## Introduction

The treat-to-target strategy is recommended to guide treatment selection for rheumatoid arthritis (RA), where the treatment target is to attain low disease activity or remission within 3 to 6 months.^1,2^ Biologics are recommended to be added to conventional synthetic disease-modifying antirheumatic drugs (csDMARDs) as first-line treatment for patients with poor prognostic factors or as second- or third-line treatment for patients with refractory disease. However, the benefit of adding biologics for a specific patient remains inaccurate, which may lead to overuse of biologics or treatment delay. Patient backgrounds are complex, and the precise effect of these backgrounds on treatment outcomes is unclear.^3,4^ Furthermore, poor prognostic factors currently used to guide treatment selection are mainly risk factors for long-term prognosis, which are crucial but lacking straightforwardness in indicating optimal treatments. Because the treat-to-target strategy aims to achieve early low disease activity, the focus of optimal treatment should initially be on improving short-term outcomes. However, despite extensive efforts, no reliable and practicable genetic or biochemical markers strongly associated with treatment response have been identified.^5,6^ On one hand, this lack of information on biochemical markers may cause overuse of biologics, leading to increased adverse effects, difficulties in treatment selection for future flares, loss of response due to antidrug antibodies, and increased medical expenditures.^7,8^ On the other, it may cause trial-and-error–based treatment selection and delay of optimal treatment.^9^

An algorithm that can estimate the accurate benefits of adding biologics over csDMARDs alone for patients with specific characteristics may help clinicians prescribe biologics only when necessary. Individual participant data meta-analysis (IPD-MA) is an evidence synthesis method that combines data from multiple randomized clinical trials (RCTs) to increase power.^10^ This type of meta-analysis can be used to build models to estimate personalized relative outcomes between 2 drugs, yet it has not been applied to construct such models for patients with RA.^11,12^ Among several proposed approaches, a 2-stage modeling approach has been previously validated on multiple sclerosis data.^13^

Our study aims to estimate the patient-specific relative outcomes of adding biologics to csDMARDs compared with csDMARDs alone. We take certolizumab as an example because it is a tumor necrosis factor α inhibitor, the most classic and widely used biologic for RA, and had plenty of IPD at the time we planned the study. To achieve this goal, we developed a model to estimate the difference in the probabilities of reaching the treatment target (ie, low disease activity or remission at 3 months) between adding certolizumab to csDMARDs and using csDMARDs alone, given the individual baseline characteristics. We fitted the model using a 2-stage approach based on IPD-MA.

## Methods

The study protocol for this meta-analysis has been registered in PROSPERO (CRD42020157595) and published.^14^ The reporting adheres to the Preferred Reporting Items for Systematic Reviews and Meta-analyses of Individual Participant Data (PRISMA-IPD) statement.^15^ As per the Ethical Guidelines for Medical and Health Research Involving Human Subjects in Japan, institutional review board approval and participant consent are not required for this study.

### Eligibility Criteria

Inclusion criteria to select studies were as follows: (1) double-blind RCTs; (2) adult (aged ≥18 years) patients with RA diagnosed by the 2010 American College of Rheumatology (ACR)/European League Against Rheumatism classification criteria^16^ or the 1987 classification criteria,^17^ with moderate to severe disease activity based on any validated composite disease activity measures; and (3) comparison of certolizumab plus any csDMARDs with placebo plus csDMARDs. We excluded studies in which patients have used certolizumab within 6 months before randomization.

### Data Collection

We searched the Cochrane CENTRAL, Scopus, MEDLINE, and World Health Organization International Clinical Trials Registry Platform from inception to March 2, 2022. The search strategy is provided in the eAppendix 1 in Supplement 1. We also searched the US Food and Drug Administration reports to identify unpublished reports. Two researchers (Y.L. and S.F.) independently screened the titles and abstracts and then the full texts of the included records and judged their eligibility. Conflicts were resolved via discussion or consultation with a third member (T.A.F.). We requested IPD for the included studies through the Vivli data-sharing platform. The following variables were provided at the individual participant level.

### Outcomes

The primary outcome was low disease activity or remission at 3 months, which was the recommended treatment target by guidelines for practice^1,2^ and clinical trials.^18^ This outcome was defined when any 1 of the following thresholds was reached^19^: Disease Activity Score based on the evaluation of 28 joints of 3.2 or lower,^20^ Clinical Disease Activity Index of 10 or lower,^21^ or Simplified Disease Activity Index of 11 or lower.^22^ We defined 3 secondary outcomes: (1) response, defined as a 50% improvement based on ACR core set variables (ACR50) at 3 months^23^; (2) severe adverse events, defined by the study investigators; and (3) infection-related adverse events, defined by the study investigators.

### Possible Risk Factors of the Outcome and Modifiers of the Relative Treatment Effect

We had prespecified candidate covariates in the protocol.^14^ Among these variables, 22 were measured and individual-level data were provided by most included studies: age; sex; body mass index (calculated as weight in kilograms divided by height in meters squared); RA history and comorbidities, including duration from onset, previous or concurrent presence of rheumatoid nodules, previous or concurrent vasculitis or neuropathy, previous use of csDMARDs other than methotrexate, previous use of biologics, concurrent use of nonsteroidal anti-inflammatory drugs, concurrent use of steroids, and concurrent use of csDMARDs other than methotrexate; RA symptoms at baseline, including severity of morning stiffness, tender joint count, swollen joint count, patient’s global assessment, physician’s global assessment, patient’s assessment of pain, patient’s assessment of fatigue, and Health Assessment Questionnaire–Disability Index function assessment; erythrocyte sedimentation rate; C-reactive protein; and rheumatoid factor. Two researchers (Y.L. and S.F.) independently assessed the risk of bias in the primary outcome according to the Cochrane Risk of Bias 2 tool.^24^

### Statistical Analysis

Before the analysis, IPD data integrity was checked by comparing the number of patients and the summary estimates of some important variables based on IPD to the reported values in the publications.

First, we conducted a 1-stage bayesian hierarchical IPD-MA to estimate the mean odds ratio (OR) between certolizumab and placebo for all the outcomes.^25^ In this model, we assumed the intercept to be independent across trials and the relative outcomes between certolizumab and placebo (ie, coefficient of the treatment parameter) to be exchangeable across trials. Details are given in eAppendix 2 in Supplement 1.

Second, we fitted a 2-stage model as described in Chalkou et al^13^ for the primary outcome low disease activity or remission. In the first stage, we fitted a logistic regression model to estimate the probability of low disease activity or remission at 3 months using baseline characteristics regardless of treatment (hereafter referred to as baseline expected probability of the outcome). We estimated the minimum sample size required to minimize overfitting and ensure precise estimation if all 22 available covariates were included in the model.^26^ We considered 3 models that used different shrinkage and variable selection approaches. The models were fitted on all trial participants, ignoring their subsequent treatment following the Predictive Approaches to Treatment Effect Heterogeneity (PATH) recommendations.^27,28^ We used the multiple imputation chained equation method to handle missing values in the covariates and outcomes.^29,30^ We proceeded to the second stage with the baseline expected probability of the outcome estimated from the model with the largest bootstrap optimism-corrected area under the curve (AUC) (ie, the optimal stage 1 model was selected based on internal validation performance). Details for the methodological steps taken at the first stage are presented in eAppendix 2 in Supplement 2.

In stage 2, we fitted a bayesian IPD meta-regression model using the baseline expected probability of the outcome estimated at stage 1 as a prognostic factor and an effect modifier. To make estimations for a new patient, we first estimated the patient’s baseline expected probability of the outcome according to the baseline characteristics. Second, we estimated the difference in the risks between receiving certolizumab plus csDMARDs and placebo plus csDMARDs. Methodological details at stage 2 are presented in eAppendix 2 in Supplement 1.

We planned to build the model only for the primary outcome in the protocol. However, the relative improvement from baseline could be an important outcome for treatment selection, especially for patients with high disease activity at baseline. Therefore, we fitted a model for the secondary outcome of ACR50 response using the same modeling approach and variables as a post hoc analysis.

We used R software, version 4.1.2 (R Foundation for Statistical Computing) for all the analyses.^31^ The stage 1 model was performed using frequentist methods established CRAN packages and self-programmed routines (see eAppendix 2 in Supplement 1 for details). The second stage was fitted in a bayesian framework using JAGS and its R interface. A normal distribution N(0, 1000) was used as the prior distribution for all mean parameters, and a half-normal prior N(0, 1) was used for all the heterogeneity parameters.^32^ We simulated 2 chains of 100 000 samples, discarded the first 10 000 samples, and thinned for every 2 samples, and the appropriateness was checked using the chain convergence trace plot. To assist decision-making, the final output is implemented in an R shiny web application.^33^ All R codes are available in github.^34^

## Results

We screened 1149 records identified by the search and found 11 eligible studies (eFigure 1 in Supplement 1). Among them, we could not access the individual-level data for 6 studies.^35,36,37,38,39,40^ Hence, we included 5 studies for analysis: RAPID (Rheumatoid Arthritis Prevention of Structural Damage) 1,^41^ RAPID 2,^42^ REALISTIC (Certolizumab Pegol for the Treatment of Patients With Active Rheumatoid Arthritis),^43^ Choy et al,^44^ and C-EARLY (Multi-center, Randomized, Double-blind, Placebo-controlled Study to Evaluate the Efficacy and Safety of Certolizumab Pegol in Combination With Methotrexate in the Treatment of Disease Modifying Antirheumatic Drugs–Naive Adults With Early Active Rheumatoid Arthritis).^45^ In total, 3790 patients (2996 female [79.1%] and 794 male [20.9%]; mean [SD] age, 52.7 [12.3] years; racial categories not provided by the data contributor) were randomized, among whom 2912 were assigned to certolizumab plus csDMARDs and 878 patients to placebo plus csDMARDs. The mean dose of certolizumab per week ranged from 100 to 200 mg. Table 1 presents the characteristics of the 5 studies. The mean Disease Activity Score ranged from 6.2 to 6.9. Patients in 4 studies^41,42,43,44^ tended to have longer disease duration compared with the other study. eTable 1 in Supplement 1 presents available characteristics of the 6 eligible studies without available IPD.^35,36,37,38,39,40^ One study^38^ had no results published, and 4 studies^35,37,39,40^ were conducted in Asian countries under similar settings with the RAPID trials. Risk-of-bias assessment for the primary outcome suggested a low risk of bias for all the included studies (eTable 2 in Supplement 1). The primary outcome was missing in 4.5% of the randomized sample (n = 169 of 3790). No serious problem was revealed in the IPD data integrity check.

**Table 1.** Characteristics of the Included Studies

Characteristic	Study					
	RAPID 1	RAPID 2	REALISTIC	Choy et al^44^	C-EARLY
No. of randomized patients	982	619	1063	247	879
Certolizumab dose, mg					
First 4 weeks	400 every 2 wk	400 every 2 wk	400 every 2 wk	400 every 4 wk	400 every 2 wk
After the first 4 weeks	400 or 200 every 2 wk	400 or 200 every 2 wk	200 every 2 wk	400 every 4 wk	200 every 2 wk
Cotreatment^a^	Methotrexate	Methotrexate	csDMARDs	Methotrexate	Methotrexate
Age, mean (SD), y	52.0 (11.6)	51.9 (11.5)	55.1 (12.5)	54.3 (12.1)	50.6 (13.5)
Duration from onset, mean (SD), y	6.2 (4.4)	6.2 (4.1)	8.7 (8.8)	9.6 (7.7)	0.2 (0.4)
BMI, mean (SD)	27.4 (5.8)	26.3 (4.6)	30.1 (7.1)	28.2 (5.9)	27.9 (5.7)
Tender joint count, mean (SD) (range, 0-28)	17.6 (6.2)	17.8 (6.4)	14.7 (6.6)	17.6 (6.5)	15.7 (6.5)
Swollen joint count, mean (SD) (range, 0-28)	14.6 (5.5)	14.4 (5.4)	11.6 (5.5)	15.4 (5.9)	12.5 (5.5)
Patient global assessment, mean (SD) (range, 0-100)	63.6 (19.5)	61.3 (20.4)	59.7 (21.8)	66.0 (13.4)	65.3 (22.0)
Physician global assessment, mean (SD) (range, 0-100)	63.8 (15.4)	64.0 (14.5)	61.5 (17.4)	71.3 (12.4)	67.7 (16.4)
Pain score, mean (SD) (range, 0-100)	63.1 (18.9)	60.9 (20.2)	54.5 (23.2)	58.7 (19.9)	66.2 (22.4)
HAQ-DI, mean (SD) (range, 0-3)	1.7 (0.6)	1.6 (0.6)	1.5 (0.6)	1.4 (0.6)	1.6 (0.6)
Fatigue score, mean (SD) (range, 0-10)	6.5 (2.0)	6.5 (1.9)	6.2 (2.2)	5.8 (2.1)	6.2 (2.2)
Baseline DAS28, mean (SD)^b^	6.9 (0.8)	6.8 (0.8)	6.4 (0.9)	6.2 (1.0)	6.7 (0.9)
CRP, median (IQR), mg/dL	1.50 (0.6-3.3)	1.6 (0.6-3.2)	0.9 (0.5-2.0)	1.1 (0.5-2.6)	1.1 (0.4-2.7)
ESR, median (IQR), mm/h	44.0 (34.0-61.0)	40.0 (33.0-51.0)	37.0 (29.0-54.5)	28.0 (16.0-41.0)	43.0 (33.0-62.0)
RF, median (IQR), IU/mL	81.0 (23.0-220.0)	58.6 (16.5-166.8)	51.7 (13.5-181.1)	49.0 (15.0-157.0)	97.0 (37.0-243.2)
Sex, No. (%)					
Female	817 (83.2)	505 (81.6)	829 (65.6)	171 (69.2)	674 (76.7)
Male	165 (16.8)	114 (18.4)	234 (34.4)	76 (30.8)	205 (23.3)
Previous use of csDMARDs, No. (%)	664 (67.6)	408 (65.9)	734 (69.0)	145 (59.2)	0 (0)
Previous use of bDMARDs, No. (%)	34 (3.5)	30 (4.8)	429 (40.4)	0 (0)	0 (0)
Low disease activity, No. (%)^c^					
Certolizumab	235 (30.3)	117 (24.8)	271 (35.2)	23 (19.0)	328 (51.2)
Placebo	8 (4.0)	3 (2.4)	32 (16.8)	6 (5.2)	79 (37.1)
ACR50, No. (%)					
Certolizumab	250 (32.1)	116 (23.7)	231 (27.7)	14 (11.6)	334 (51.9)
Placebo	13 (6.6)	5 (3.9)	22 (10.6)	2 (1.7)	87 (41.4)
SAEs, No. (%)					
Certolizumab	46 (5.9)	26 (5.3)	60 (7.1)	14 (11.1)	28 (4.2)
Placebo	7 (3.5)	3 (2.4)	15 (7.1)	10 (8.3)	7 (3.2)
Infection-related AEs, No. (%)					
Certolizumab	178 (22.7)	94 (19.1)	262 (30.8)	38 (31.2)	179 (27.1)
Placebo	36 (18.1)	24 (18.9)	50 (23.6)	18 (14.9)	47 (21.5)

Abbreviations: ACR50, 50% improvement based on American College of Rheumatology core set variables; AE, adverse event; bDMARD, biological disease-modifying antirheumatic drug; BMI, body mass index (calculated as weight in kilograms divided by height in meters squared); C-EARLY, Multi-center, Randomized, Double-blind, Placebo-controlled Study to Evaluate the Efficacy and Safety of Certolizumab Pegol in Combination With Methotrexate in the Treatment of Disease Modifying Antirheumatic Drugs–Naive Adults With Early Active Rheumatoid Arthritis; csDMARD, conventional synthetic disease-modifying antirheumatic drug; CRP, C-reactive protein; DAS28, Disease Activity Score based on the evaluation of 28 joints; ESR, erythrocyte sedimentation rate; HAQ-DI, Health Assessment Questionnaire–Disability Index; RAPID, Rheumatoid Arthritis Prevention of Structural Damage; REALISTIC, Certolizumab Pegol for the Treatment of Patients With Active Rheumatoid Arthritis; RF, rheumatoid factor; SAE, severe adverse event.

SI conversion factor: To convert CRP to milligrams per liter, multiply by 10.

^a^ Methotrexate indicates that only methotrexate could be used, whereas csDMARDs suggests that several csDMARDs were allowed according to the study definition.

^b^ The DAS28 is calculated from tender joint count (range, 0-28), swollen joint count (range, 0-28), patient global assessment of disease activity (range, 0-100), and ESR.

^c^ Low disease activity is defined as achieving low disease activity or remission based on 1 of the following scales at 3 months: DAS28, Clinical Disease Activity Index, or Simplified Disease Activity Index. Missing values in each study were as follows: RAPID 1, 10 (4.0%); RAPID 2, 22 (3.6%); REALISTIC, 103 (9.7%); Choy et al,^44^ 9 (0.9%); and C-EARLY, 25 (2.8%).

### Mean Relative Outcomes of Add-On Certolizumab by IPD-MA

eTable 3 in Supplement 1 shows the estimated mean ORs and coefficients from the bayesian IPD-MA models for 4 outcomes. The mean OR for achieving low disease activity or remission in the certolizumab plus csDMARDs group compared with placebo plus csDMARDs was 5.32 (95% credible interval [CrI], 1.85-13.89). The OR for ACR50 response was 4.74 (95% CrI, 1.85-10.94). For adverse event outcomes, adding certolizumab was associated with a higher risk of serious adverse events, but with high uncertainty (OR, 1.47; 95% CrI, 0.88-2.43), and with an increased risk of infection (OR, 1.44; 95% CrI, 1.02-2.03).

### Individual Relative Outcomes of Add-On Certolizumab by the 2-Stage Model

**Figure 1.**
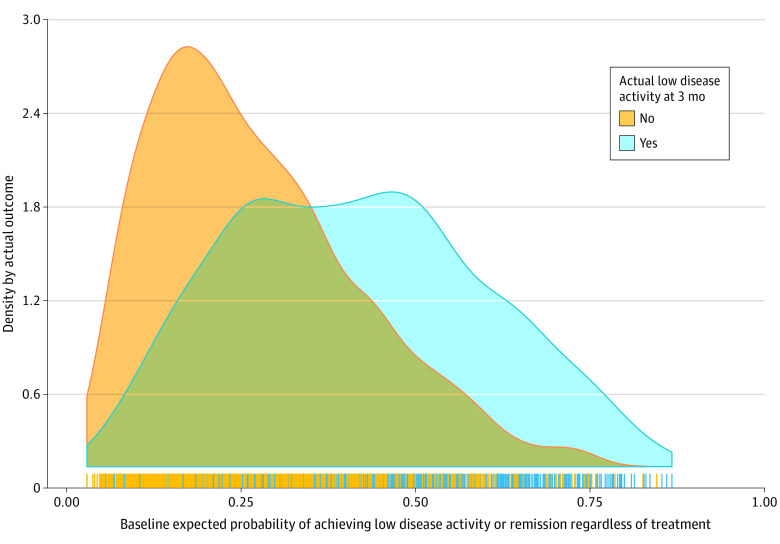
Estimated Baseline Expected Probabilities of Low Disease Activity or Remission at 3 Months From the Stage 1 Model for the Study Population

For the primary outcome of low disease activity or remission, the minimum sample size required for a model of 22 covariates was 1953, based on the assumption that the outcome event rate is 30%, and the Cox-Snell *R*^2^ is 0.1. Our sample size of 3790 exceeded the minimum required sample size. eTable 4 in Supplement 1 gives the regression coefficients and bootstrap optimism-corrected performance for all 3 models. The selected stage 1 model was a penalized maximum likelihood logistic regression that included 16 preselected variables (internal validation performance: AUC = 0.72, calibration intercept = 0.03, slope = 0.98) (eAppendix 3 in Supplement 1; apparent performance is presented in eFigure 2 in Supplement 1). Figure 1 shows the distribution of the baseline expected probabilities of low disease activity or remission despite treatment for patients who had the outcome or not separately. The estimated parameters of the stage 2 bayesian IPD meta-regression model are given in Table 2, and the diagnostic trace plots for the estimates are presented in eFigure 3 in Supplement 1. The OR for patients with a mean baseline expected probability of the outcome was 6.31 (95% CrI, 2.22-15.25). The coefficient of the interaction term between the baseline expected probability and treatment was −0.14 (95% CrI, −0.57 to 0.30), suggesting that the baseline expected probability was a prognostic factor but not a significant effect modifier.

**Table 2.** Estimated Parameters for the Stage 2 Model

Parameter	Interpretation of the parameter	Odds ratio (95% credible interval)	
		Low disease activity or remission	ACR50 response
α1	Intercept for study 1 (RAPID 1^41^)	−2.89 (−3.60 to −2.26)	−2.34 (−2.94 to −1.80)
α2	Intercept for study 2 (RAPID 2^42^)	−3.40 (−4.54 to −2.53)	−2.87 (−3.75 to −2.15)
α3	Intercept for study 3 (REALISTIC^43^)	−1.96 (−2.44 to −1.49)	−1.99 (−2.49 to −1.52)
α4	Intercept for study 4 (Choy et al^44^)	−2.96 (−3.87 to −2.17)	−3.66 (−4.90 to −2.71)
α5	Intercept for study 5 (C-EARLY^45^)	−1.35 (−1.77 to −0.95)	−1.17 (−1.74 to −0.61)
Δ	Coefficient of treatment: the log-odds average treatment effect	1.72 (0.80 to 2.72)	1.45 (0.64 to 2.35)
Odds ratio	Mean treatment effect: exp (δ)	6.31 (2.22 to 15.25)	4.70 (1.90 to 10.52)
γ_0_	Coefficient of baseline expected probability of the outcome	1.07 (0.66 to 1.47)	1.02 (0.34 to 1.74)
Γ	Coefficient of the interaction of baseline expected probability and treatment: effect modification of the baseline expected probability on the outcome	−0.14 (−0.57 to 0.30)	−0.15 (−0.89 to 0.61)
T	Heterogeneity in the treatment effect δ across studies	0.91 (0.38 to 1.80)	0.77 (0.23 to 1.64)
σγ_0_	Heterogeneity in γ_0_ across studies	0.18 (0.01 to 0.66)	0.26 (0.01 to 0.90)
Σγ	Heterogeneity in γ across studies	0.20 (0.01 to 0.71)	0.28 (0.01 to 0.94)

Abbreviations: ACR50, 50% improvement based on American College of Rheumatology core set variables; C-EARLY, Multi-center, Randomized, Double-blind, Placebo-controlled Study to Evaluate the Efficacy and Safety of Certolizumab Pegol in Combination With Methotrexate in the Treatment of Disease Modifying Antirheumatic Drugs–Naive Adults With Early Active Rheumatoid Arthritis; RAPID, Rheumatoid Arthritis Prevention of Structural Damage; REALISTIC, Certolizumab Pegol for the Treatment of Patients With Active Rheumatoid Arthritis.

**Figure 2.**
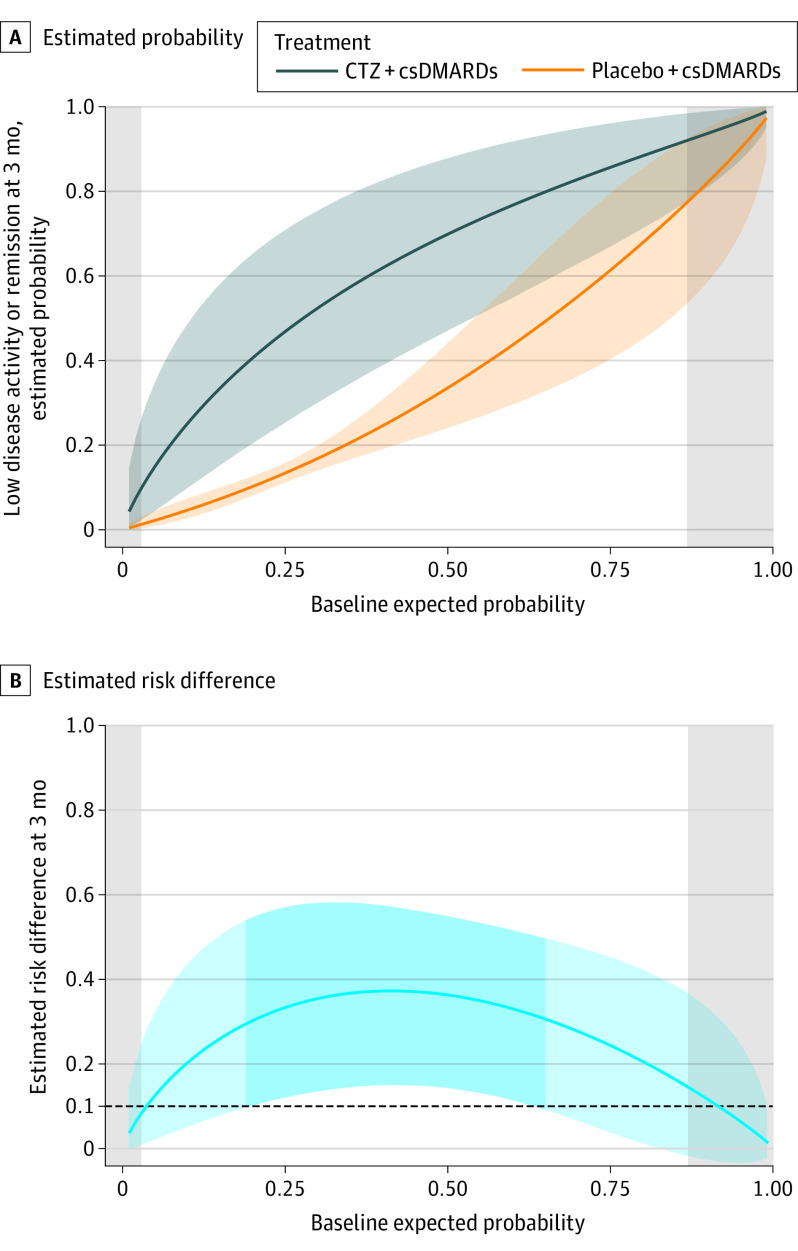
Estimated Individual Relative Outcome of Add-On Certolizumab by the 2-Stage Model for New Patients Given the Baseline Expected Probability A, Estimated probability of low disease activity or remission, if receiving certolizumab plus conventional synthetic disease-modifying antirheumatic drugs (csDMARDs) or placebo plus csDMARDs, given the baseline expected probability of the outcome. Shaded areas indicate 95% credible intervals (CrIs). B, Estimated risk difference between the 2 groups. The estimated stage 1 baseline expected probabilities for all the participants in our study range from 2.88% to 86.81%. The light shaded areas indicate the 95% CrI for risk difference, and the dark shaded area indicates patients whose baseline expected probability is between 20% and 65% (lower 95% CrI boundary of risk difference is >10%).

Figure 2A and B displays the estimated probabilities of reaching low disease activity or remission for the 2 treatments and their risk difference for new patients with given baseline expected probabilities. Adding certolizumab to csDMARDs appears to be better than csDMARDs alone regardless of baseline expected probabilities. Patients with a baseline expected probability between 20% and 65% will probably benefit from adding certolizumab because the lower 95% CrI boundary of the estimated risk difference is larger than 10%. On the other hand, there is uncertainty about the benefit for patients with either very high or very low baseline expected probability.

For the secondary outcome of ACR50 response, the results of the stage 1 model are given in eTable 4 and eFigure 4 in Supplement 1. The results from the stage 2 model are given in Table 2 and eFigure 5 in Supplement 1. eFigure 6 in Supplement 1 is a screenshot of the R shiny web app. After inputting 16 baseline characteristics, a bar plot will depict the estimated probabilities of low disease activity or remission on certolizumab plus csDMARDs or csDMARDs alone.

## Discussion

To our knowledge, this is the first model to estimate the relative benefit of adding biologics to RA treatment, using certolizumab as an example. The model was fitted using a 2-stage approach based on the IPD of 3790 patients from 5 large RCTs of low risk of bias. In stage 1, a penalized maximum likelihood logistic regression that included 16 preselected variables was chosen, with a bootstrap optimism-corrected AUC of 0.72. This model estimated the baseline expected probability of the outcome regardless of treatment, which was then used as a covariate in the stage 2 meta-regression model to estimate the difference in the risks of adding certolizumab over csDMARDs alone. Finally, we made a shiny web application to visualize the estimated results interactively. The model indicates that adding biologics to csDMARDs was associated with a higher probability of reaching the short-term treatment target in general. However, the benefit was limited for patients with very low or high baseline expected probability.

In attempting to develop a model that can assist treatment selection, we selected low disease activity or remission at 3 months as the outcome. This outcome is the recommended common target for patients at any stage,^1,2^ making it more straightforward to guide drug selection compared with long-term structural or functional outcomes. Moreover, although many existing models were based on observational data to estimate the outcome for a particular treatment,^3,46,47^ our model was based on RCT data aiming to estimate the difference between 2 treatment approaches, which we consider more crucial in treatment selection.

As expected, our model suggests that adding certolizumab was associated with an increased probability of achieving the treatment target for all patients. However, the benefit seems to be limited to patients whose baseline expected probability exceeds 65%. Considering potential adverse effects, cost, and future flare management, csDMARDs alone could be a reasonable choice, which is consistent with guideline recommendations.^2^ Similarly, patients with a baseline expected probability below 20% may find limited benefits from adding certolizumab, and alternative outcomes, such as relative improvements from baseline (eg, ACR50 response), could guide drug selection. In addition, there is chance of misclassification of disease status in patients with persistently high disease activity scores because the subjective components in disease activity measures may lead to discordance with the clinically judged activity.^48,49^ For these patients, differentiating between persistent inflammation and subjective reporting without evidence of inflammation is critical.^50^ For patients with enduring inflammation, prescribing medications with different targets, increasing the dose, or adding other drugs experimentally may be viable options. Conversely, patients reporting severe symptoms without evidence of inflammation may benefit from symptomatic treatments.

### Limitations

Our study has limitations. First, several potential risk factors, such as smoking, genetic or biochemical markers, and structural damage, were not available in the IPD data set. Neither could we include precise information for particular variables, such as the number of previous treatment attempts and their responses, which could have impacted the model performance. Second, the 2-stage approach could not identify individual prognostic factors and effect modifiers, which may pose interpretation challenges. Nevertheless, it may mitigate overfitting problems compared with the 1-stage modeling approach.^51^ Moreover, because the stage 1 risk model can be replaced by alternative models, future studies with ample sample sizes can explore advanced machine learning methods.^46,47^ In addition, although a bayesian framework could have been used at stage 1, we adopted a frequentist framework to leverage available penalization options in existing R packages. This approach might have led to overly precise estimates in stage 2, because it might not fully capture uncertainty from stage 1. Nonetheless, it is unlikely that a bayesian setting with noninformative priors would produce different results from the frequentist setting. Third, of the 11 eligible studies, only 5 studies^41,42,43,44,45^ provided IPD, potentially cause data availability bias. Caution should be exercised when applying the model to Asian patients because most unavailable studies were conducted in Asia, where certolizumab was marketed by different companies. Fourth, although we performed internal validation for the stage 1 model, we could not validate the estimated relative benefit between 2 groups from the stage 2 model because of lack of established validation methods (eAppendix 4 in Supplement 1). Future external validation of the benefit estimation is necessary. In addition, there are unresolved essential issues that require future research. For instance, our use of certolizumab as an example of biologics was due to its abundant IPD availability during the planning stage, but it may not fully represent all biologics. We are now planning to build a model based on an IPD network meta-analysis to compare multiple biologics simultaneously.

## Conclusions

In this meta-analysis, we have presented a model that estimates the patient-specific relative benefit of adding biologics to csDMARDs for RA, using certolizumab as an example. A shiny application was created to present the results interactively, assisting patient-centered treatment selection in accordance with the treat-to-target strategy. Adding biologics was associated with an increased probability of reaching the treatment target for patients with moderate baseline risks. However, the benefit was limited for patients with very low or high baseline risks, for whom other evaluations may be necessary.

## Notes

Supplement 1. **eFigure 1.** Flow Chart of Study Selection **eFigure 2.** ROC Curves and Calibration Plots for the Selected Stage-One Models (Apparent Performance) **eFigure 3.** Chain Convergence Trace Plots of the Parameters for the Stage-Two Model of the Primary Outcome **eFigure 4.** Estimated Baseline Expected Probabilities of Achieving ACR50 Response From Stage-One Model for the Study Population **eFigure 5.** Estimated Individual Relative Outcome (ACR50 Response) of Add-On CTZ by the Two-Stage Model for New Patients Given the Baseline Expected Probability **eFigure 6.** A Screenshot of the R Shiny Web Application to Display the Estimated Results for Individual Patients **eTable 1.** Characteristics of the Six Studies Whose IPD Was Inaccessible **eTable 2.** Risk of Bias in the Five Included Studies for the Primary Outcome **eTable 3.** Average Relative Outcomes (Estimated From the Bayesian IPD-MA Model) **eTable 4.** Estimated Parameters for the Stage-One Models **eAppendix 1.** Search Strategy **eAppendix 2.** Details of the Statistical Analysis **eAppendix 3.** Variable Selection Based on Previous Literature **eAppendix 4.** Variable Preprocessing

Supplement 2.
Data Sharing Statement
