# Supplementary material for: Estimating Patient-Specific Relative Benefit of Adding Biologics to Conventional Rheumatoid Arthritis Treatment: An Individual Participant Data Meta-Analysis
Source: JAMA Netw Open. 2023 Jun 30;6(6):e2321398. doi: 10.1001/jamanetworkopen.2023.21398 (PMC10314313; doi:10.1001/jamanetworkopen.2023.21398)
Supplement: Supplement 1. — eFigure 1. Flow Chart of Study Selection eFigure 2. ROC Curves and Calibration Plots for the Selected Stage-One Models (Apparent Performance) eFigure 3. Chain Convergence Trace Plots of the Parameters for the Stage-Two Model of the Primary Outcome eFigure 4. Estimated Baseline Expected Probabilities of Achieving ACR50 Response From Stage-One Model for the Study Population eFigure 5. Estimated Individual Relative Outcome (ACR50 Response) of Add-On CTZ by the Two-Stage Model for New Patients Given the Baseline Expected Probability eFigure 6. A Screenshot of the R Shiny Web Application to Display the Estimated Results for Individual Patients eTable 1. Characteristics of the Six Studies Whose IPD Was Inaccessible eTable 2. Risk of Bias in the Five Included Studies for the Primary Outcome eTable 3. Average Relative Outcomes (Estimated From the Bayesian IPD-MA Model) eTable 4. Estimated Parameters for the Stage-One Models eAppendix 1. Search Strategy eAppendix 2. Details of the Statistical Analysis eAppendix 3. Variable Selection Based on Previous Literature eAppendix 4. Variable Preprocessing [file jamanetwopen-e2321398-s001.pdf]

Supplementary Online Content

Luo Y, Chalkou K, Funada S, Salanti G, Furukawa TA. Estimating patient-specific relative benefit of adding biologics to conventional rheumatoid arthritis treatment: an individual participant data meta-analysis. *JAMA Netw Open*. 2023;6(6):e2321398. doi:10.1001/jamanetworkopen.2023.21398

eFigure 1. Flow Chart of Study Selection
eFigure 2. ROC Curves and Calibration Plots for the Selected Stage-One Models (Apparent Performance)
eFigure 3. Chain Convergence Trace Plots of the Parameters for the Stage-Two Model of the Primary Outcome
eFigure 4. Estimated Baseline Expected Probabilities of Achieving ACR50 Response From Stage-One Model for the Study Population
eFigure 5. Estimated Individual Relative Outcome (ACR50 Response) of Add-On CTZ by the Two-Stage Model for New Patients Given the Baseline Expected Probability
eFigure 6. A Screenshot of the R Shiny Web Application to Display the Estimated Results for Individual Patients
eTable 1. Characteristics of the Six Studies Whose IPD Was Inaccessible
eTable 2. Risk of Bias in the Five Included Studies for the Primary Outcome
eTable 3. Average Relative Outcomes (Estimated From the Bayesian IPD-MA Model)
eTable 4. Estimated Parameters for the Stage-One Models
eAppendix 1. Search Strategy
eAppendix 2. Details of the Statistical Analysis
eAppendix 3. Variable Selection Based on Previous Literature
eAppendix 4. Variable Preprocessing

This supplementary material has been provided by the authors to give readers additional information about their work.

© 2023 Luo Y et al. *JAMA Network Open.*

eFigure 1. Flow Chart of Study Selection

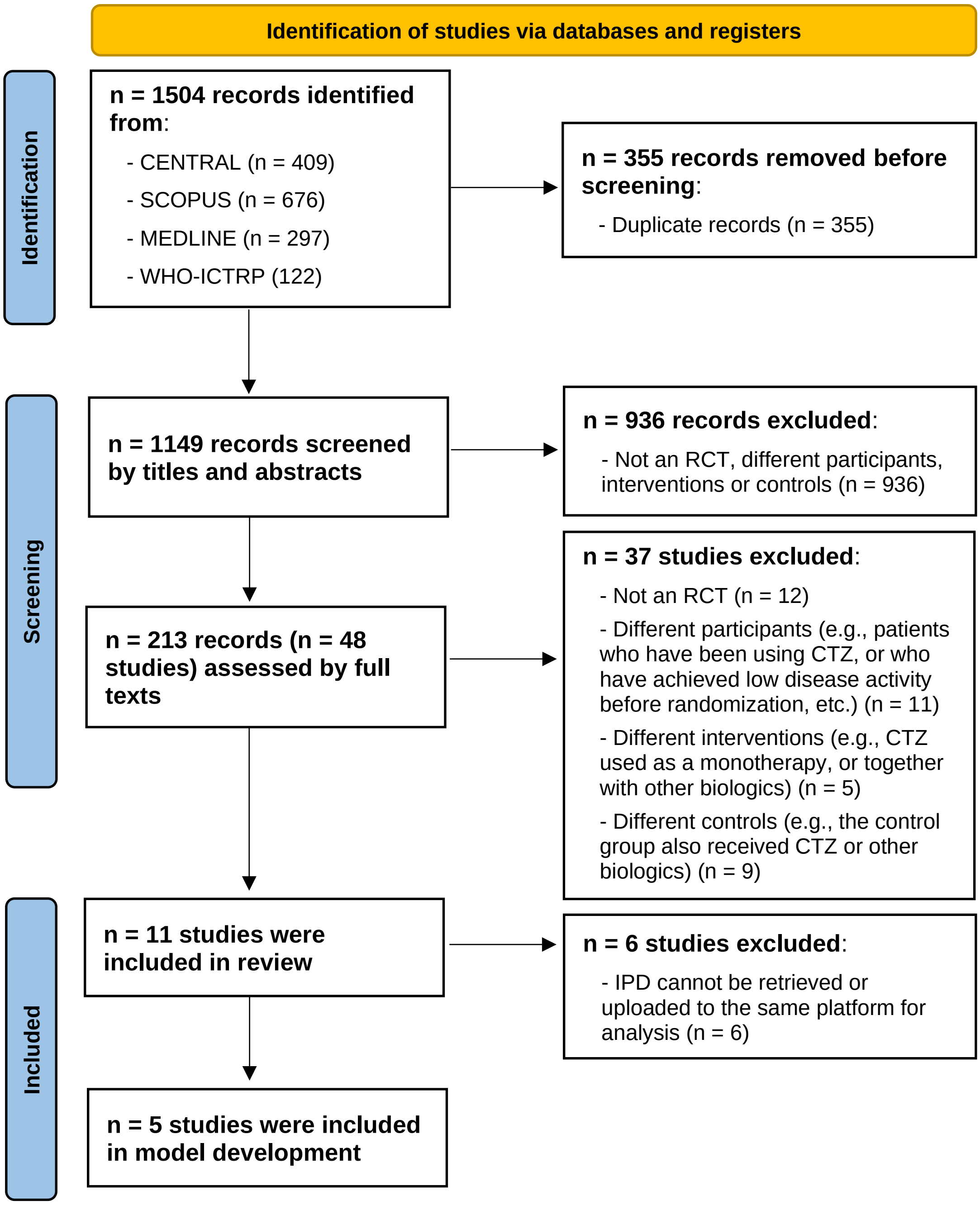

Abbreviations: RCT – randomized controlled trial; CTZ – certolizumab; IPD – individual participant data.

© 2023 Luo Y et al. *JAMA Network Open.*

eFigure 2. ROC Curves and Calibration Plots for the Selected Stage-One Models (Apparent Performance)

(A) ROC for low disease activity model.

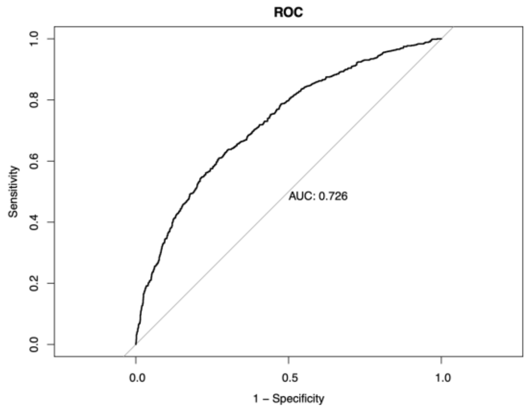

(B) Calibration plot for low disease activity model.

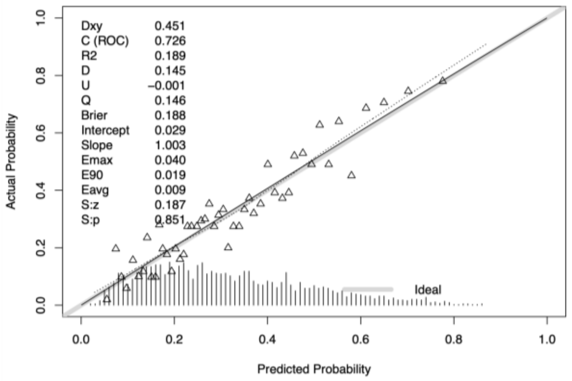

(C) ROC forACR50 model.

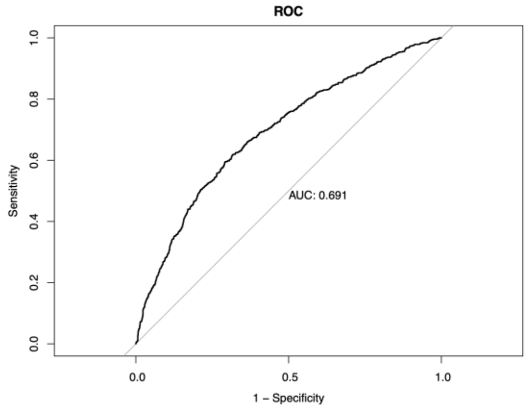

(D) Calibration plot for ACR50 model.

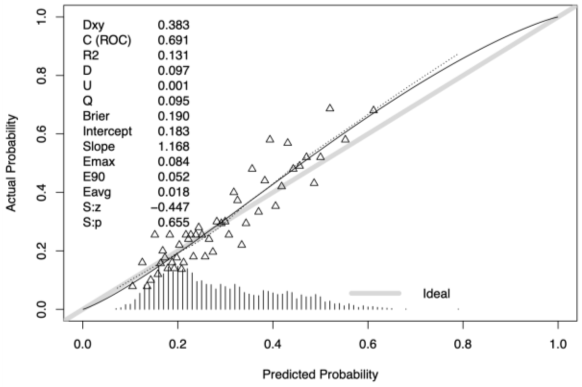

© 2023 Luo Y et al. *JAMA Network Open.*

eFigure 3. Chain Convergence Trace Plots of the Parameters for the Stage-Two Model of the Primary Outcome

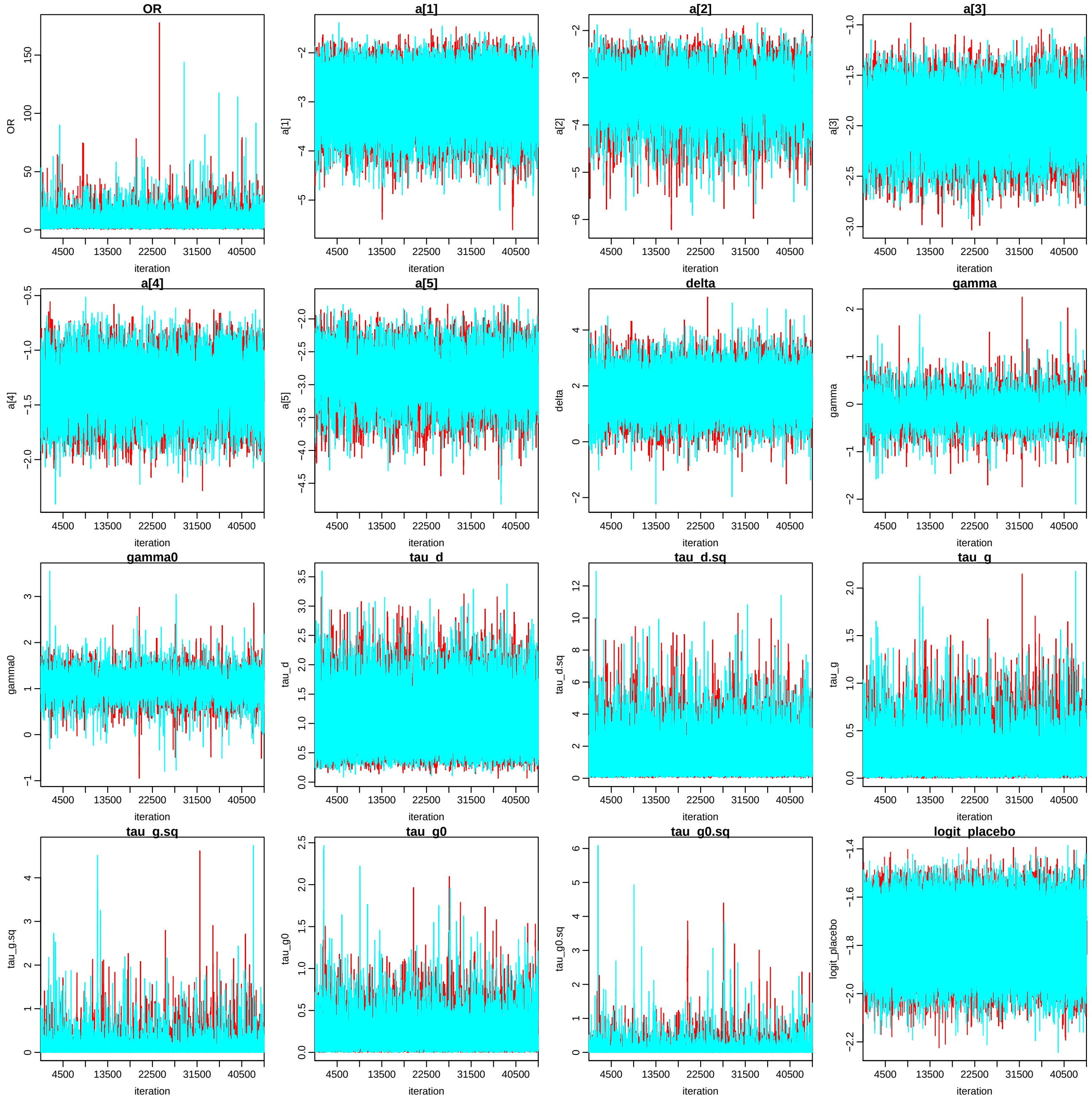

© 2023 Luo Y et al. *JAMA Network Open.*

eFigure 4. Estimated Baseline Expected Probabilities of Achieving ACR50 Response From Stage-One Model for the Study Population

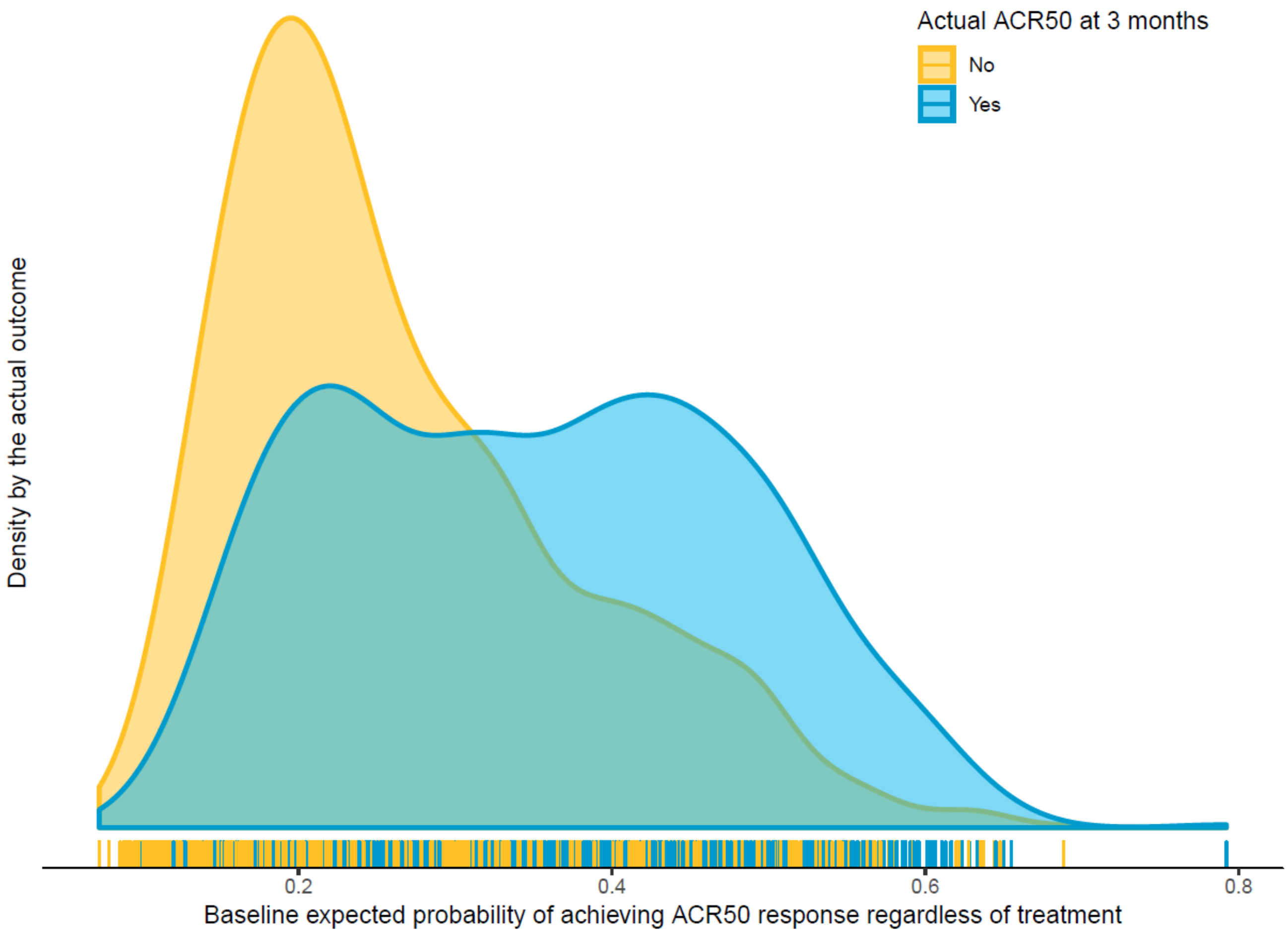

© 2023 Luo Y et al. *JAMA Network Open.*

eFigure 5. Estimated Individual Relative Outcome (ACR50 Response) of Add-On CTZ by the Two-Stage Model for New Patients Given the Baseline Expected Probability

(A) Estimated probability of ACR50 response, if receiving CTZ+csDMARDs or placebo+csDMARDs respectively, given the baseline expected probability of the outcome; (B) Estimated risk difference between the two groups.

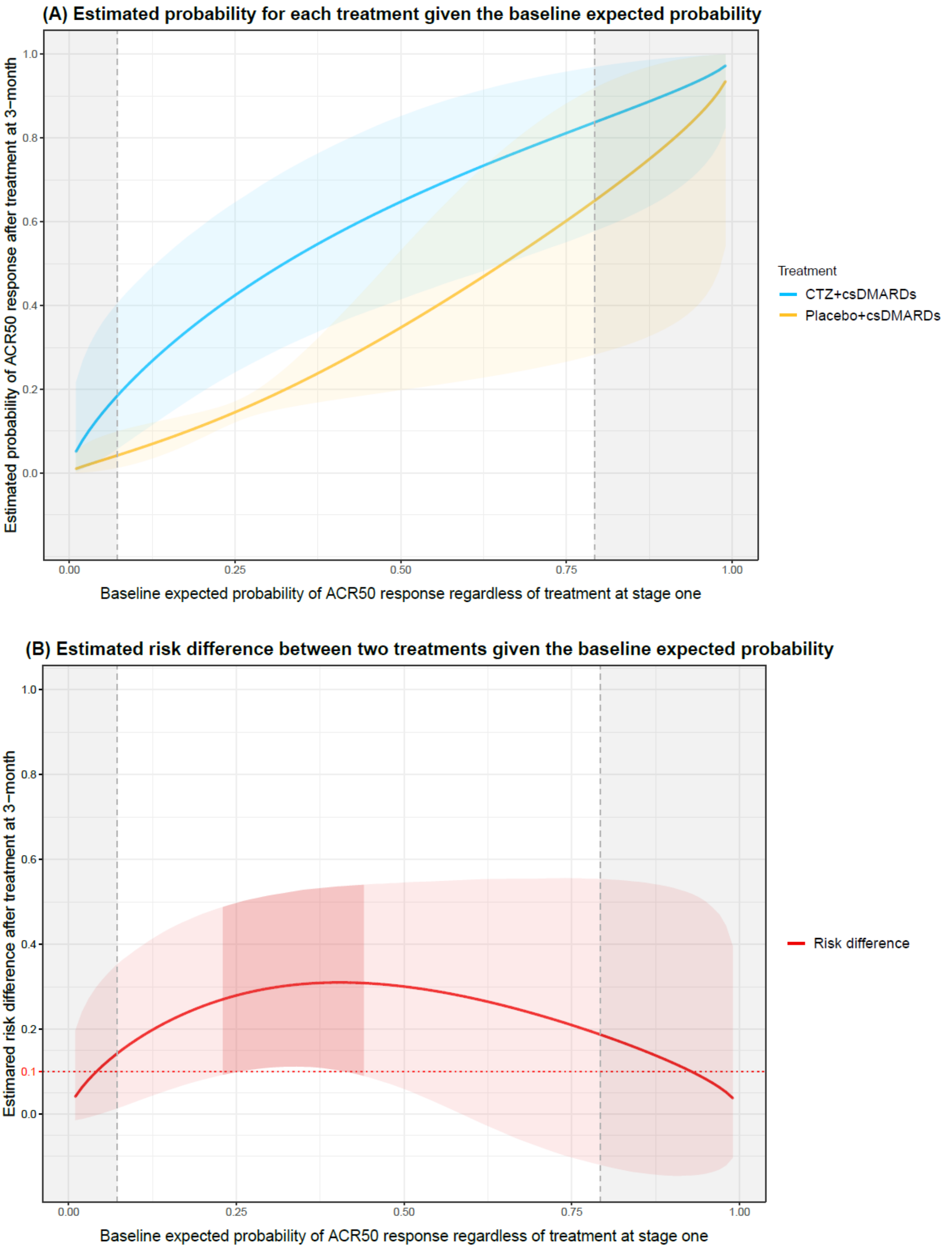

Abbreviations: CTZ: certolizumab; csDMARD: conventional synthetic disease modifying anti-rheumatic drug. The Estimated stage-one baseline expected probabilities for all the participants in our study range from 7.27% to 79.22%, which is shown in white background. The light red area indicates the 95% CrI for risk difference, the dark red area indicates patients whose baseline expected probability is between 23% to 44% (lower 95% CrI boundary of risk difference is larger than 10%).

© 2023 Luo Y et al. *JAMA Network Open.*

eFigure 6. A Screenshot of the R Shiny Web Application to Display the Estimated Results for Individual Patients

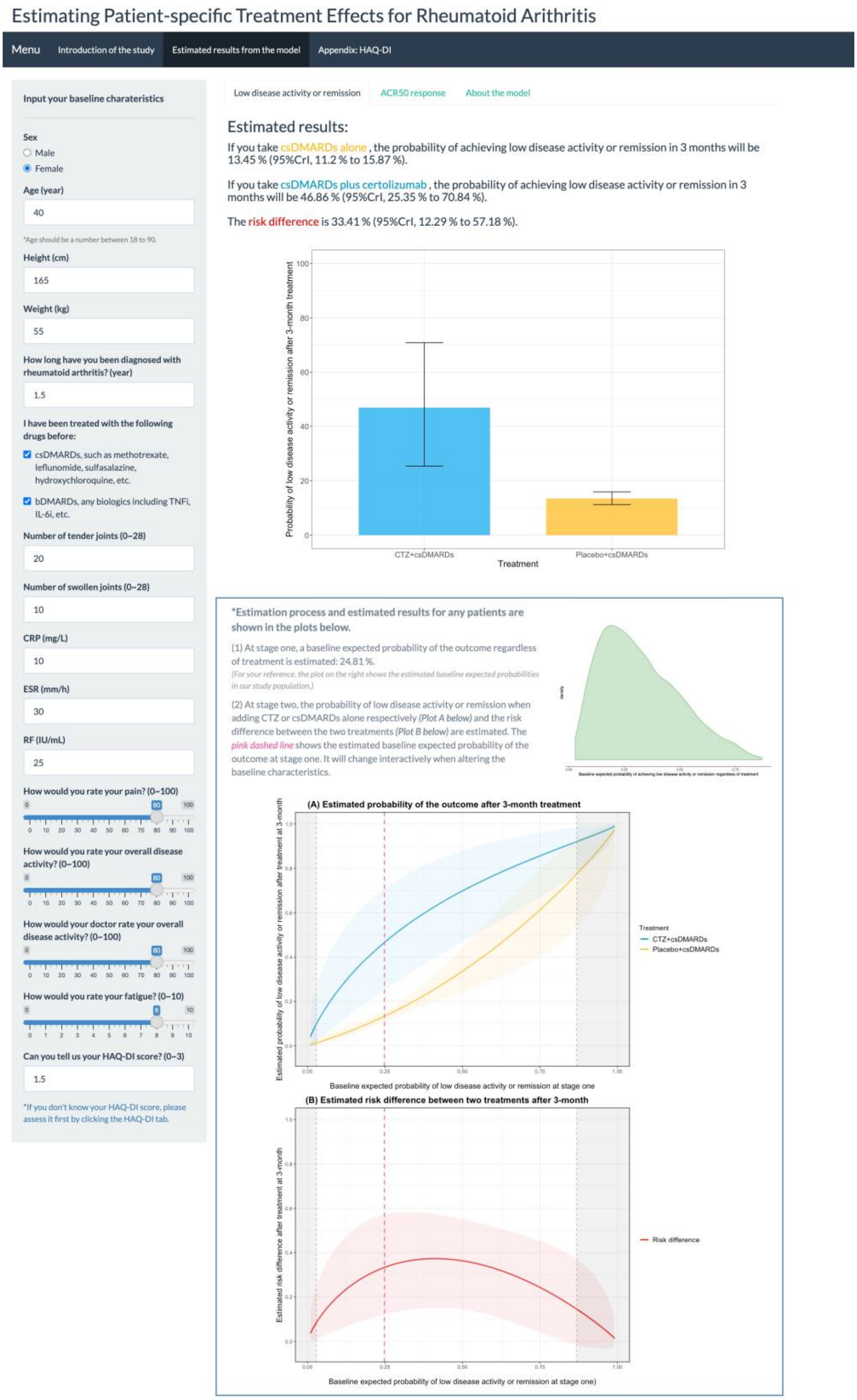

eTable 1. Characteristics of the Six Studies Whose IPD Was Inaccessible

	Study, Mean (SD)					
Characteristics	C-OPERA	J-RAPID	Kang-2018	RAPID-C	Schiff-2014	PreCePRA
Country	Japan	Japan	Korea	China	US	Germany
Randomized patients	316	316	127	430	37	Protocol
CTZ dose (mg)						
First 4w	400 every 2w	200 or 400 every 2w	400 every 2w	400 every 2w	400 every 2w	400 every 2w
After first 4w	200 every 2w	100, 200 or 400 every 2w	200 every 2w	200 every 2w	200 every 2w	200 every 2w
Co-treatment [a]	MTX	MTX	MTX	MTX	csDMARDs	csDMARDs
Age (years)	49.2 (10.5)	53.1 (10.9)	51.3 (11.5)	47.9 (11.6)	56.9 (not reported)	Protocol
Duration from onset (years)	0.35 (0.2)	5.8 (4.1)	6.2 (4.3)	6.9 (6.7)	12.5 (not reported)	Protocol
BMI, kg/m^2	22.4 (3.4)	Not reported	22.9 (3.2)	22.4 (3.5)	Not reported	Protocol
Tender joint count (0~28)	8.6 (6.3)	Not reported	Not reported	Not reported	Not reported	Protocol
Swollen joint count (0~28)	8.3 (5.3)	Not reported	Not reported	Not reported	Not reported	Protocol
Patient global assessment (0~100)	51.6 (22.5)	55.6 (21.0)	61.4 (21.1)	Not reported	Not reported	Protocol
Physician global assessment (0~100)	57.5 (21.0)	62.5 (16.8)	64.5 (16.3)	Not reported	Not reported	Protocol
HAQ-DI (0~3)	1.0 (0.7)	1.1 (0.7)	1.5 (0.7)	Not reported	1.4 (not reported)	Protocol
Baseline DAS28	5.4 (1.2)	6.3 (0.8)	7.4 (1.2)	6.7 (1.0)	5.5 (not reported)	Protocol
Sex, No. (%)						Protocol
Men	60 (19.0)	54 (17.1)	14 (11.6)	67 (15.6)	Not reported	Protocol
Women	256 (81.0)	262 (82.9)	107 (88.4)	363 (84.4)	Not reported	Protocol
Previous treatments	19.0% had treatment experience other than MTX	13.6% had used TNFi than CTZ	Not reported	20.9% had used TNFi	Had previous secondary inadequate response or were intolerant to a TNFi other than CTZ	Protocol
Outcome at week 12, No. (%)	Not reported	Not reported	ACR50: CTZ: 35 (43.2) PBO: 8 (20.0)	Not reported	Not reported	Protocol

Abbreviations: CTZ: certolizumab; PBO: placebo; MTX: methotrexate; csDMARD: conventional synthetic disease modifying anti-rheumatic drug; BMI: body mass index; CRP: C-reactive protein; ESR: erythrocyte sedimentation rate; RF: rheumatoid factor; HAQ-DI: Health Assessment Questionnaire-Disability Index; DAS: disease activity score; SD: standard deviation.

[a] MTX indicates that only MTX could be used, while csDMARDs suggests that several csDMARDs were allowed according to the study definition.

© 2023 Luo Y et al. *JAMA Network Open.*

eTable 2. Risk of Bias in the Five Included Studies for the Primary Outcome

**Study**	Randomization process	Deviations from the intended interventions	Missing outcome data	Measurement of the outcome	Selection of the reported results	**Overall risk of bias**
**RAPID1**	●	●	●	●	●	●
**RAPID2**	●	●	●	●	●	●
**REALISTIC**	●	●	●	●	●	●
**Choy-2012**	●	●	●	●	●	●
**C-EARLY**	●	●	●	●	●	●

●: Low risk of bias; ●: Some concerns; ●: High risk of bias.

© 2023 Luo Y et al. *JAMA Network Open.*

eTable 3. Average Relative Outcomes (Estimated From the Bayesian IPD-MA Model)

	**Primary outcome: low disease activity or remission**	**Secondary outcome: ACR50**	**Secondary outcome: SAEs**	**Secondary outcome: infection AEs**
**OR** (average treatment effect: exp(δ))	**5.32 (1.85 to 13.89)**	**4.74 (1.85 to 10.94)**	**1.47 (0.88 to 2.43)**	**1.44 (1.02 to 2.03)**
$\boldsymbol{\alpha_1}$ (intercept for study 1 [RAPID1])	-3.08 (-3.80 to -2.47)	-2.61 (-3.18 to -2.11)	-3.22 (-3.83 to -2.71)	-1.54 (-1.84 to -1.25)
$\boldsymbol{\alpha_2}$ (intercept for study 2 [RAPID2])	-3.46 (-4.53 to -2.62)	-3.09 (-3.96 to -2.37)	-3.41 (-4.22 to -2.81)	-1.63 (-1.98 to -1.23)
$\boldsymbol{\alpha_3}$ (intercept for study 3 [REALISTIC])	-1.65 (-2.04 to -1.28)	-2.18 (-2.62 to -1.76)	-2.76 (-3.22 to -2.28)	-1.17 (-1.44 to -0.91)
$\boldsymbol{\alpha_4}$ (intercept for study 4 [Choy-2012])	-2.98 (-3.81 to -2.27)	-3.92 (-5.15 to -2.96)	-2.44 (-3.00 to -1.93)	-1.52 (-1.98 to -1.14)
$\boldsymbol{\alpha_5}$ (intercept for study 5 [C-EARLY])	-0.56 (-0.84 to -0.29)	-0.39 (-0.67 to -0.11)	-3.46 (-4.05 to -2.93)	-1.32 (-1.59 to -1.05)
$\boldsymbol{\delta}$ (summary estimate of the log-odds treatment effect)	1.55 (0.62 to 2.56)	1.45 (0.61 to 2.39)	0.35 (-0.13 to 0.89)	0.35 (0.02 to 0.71)
$\boldsymbol{\tau}$ (heterogeneity across studies)	0.93 (0.42 to 1.81)	0.83 (0.35 to 1.70)	0.30 (0.01 to 0.99)	0.23 (0.01 to 0.80)

Abbreviations: OR: odds ratio; SAE: severe adverse event; AE: adverse event.
All the results are shown in point estimate and 95% credible interval.

© 2023 Luo Y et al. *JAMA Network Open.*

eTable 4. Estimated Parameters for the Stage-One Models

Parameters[a]	Primary outcome: low disease activity or remission				Secondary outcome (ACR50): LR16+PML
	(1) LR22+PML	(2) LR22+LASSO[b]		(3) LR16+PML	
		λ_MAX	λ_1SE		
**Intercept**	5.0518	5.1253	1.4458	**5.2217**	2.5682
**Age**[c]	-0.1322	-0.1061	0	**-0.1807**	-0.2431
**Sex** (0=female, 1=male)	-0.0964	-0.1022	0	**-0.0242**	-0.1148
**BMI** (kg/m^2)	-0.0252	-0.0270	-0.0034	**-0.0258**	-0.0255
**Duration from onset**[c] (years)	-0.1893	-0.2135	-0.1495	**-0.1873**	-0.2234
**Rheumatoid nodules** (0/1)	-0.0449	-0.0157	0	-	-
**Vasculitis** (0/1)	-0.3552	-0.3474	0	-	-
**Previous use of csDMARDs** (0/1)	-0.3553	-0.3439	-0.0622	**-0.3881**	-0.2483
**Concurrent use of csDMARDs other than MTX** (0/1)	0.2484	0.2561	0	-	-
**Previous use of bDMARDs** (0/1)	0.2299	0.2683	0	**0.1245**	0.1511
**Concurrent use of NSAIDs** (0/1)	-0.0849	-0.0709	0	-	-
**Concurrent use of steroids** (0/1)	-0.0616	-0.0445	0	-	-
**Tender joint count**[c] (0-28)	-0.2411	-0.2624	-0.2452	**-0.2508**	-0.0762
**Swollen joint count** (0-28)	-0.0301	-0.0291	-0.0137	**-0.0335**	0.0034
**Morning stiffness** (0-5)	-0.0658	-0.0734163	0	-	-
**Patient global assessment** (0-100)	-0.0019	-0.0016	-0.0010	**-0.0027**	0.0017
**Physician global assessment** (0-100)	-0.0060	-0.0061	-0.0023	**-0.0060**	-0.0009
**Pain** (0-100)	-0.0016	-0.0015	0	**-0.0020**	0.0035
**CRP**[c] (mg/L)	-0.0328	-0.0248	0	**-0.0663**	0.0514
**ESR** (mm/h)	-0.0054	-0.0061	0	**-0.0043**	-0.0027
**RF**[c] (IU/mL)	-1.5855	-1.6407	0	**-1.4920**	-1.3997
**HAQ** (0-3)	-0.1985	-0.2055	-0.1589	**-0.2367**	-0.1445
**Fatigue** (0-10)	-0.0408	-0.0389	-0.0214	**-0.0419**	-0.0652
**Bootstrap optimism corrected performance of each model**					
**AUC**	0.7193	0.7198	0.7136	0.7203	0.6836
**Calibration intercept**	0.0103	0.0090	0.0382	0.0254	0.0475
**Calibration slope**	1.0101	0.9699	1.5037	0.9751	1.1222

Abbreviations: BMI: body mass index; csDMARD: conventional synthetic disease modifying anti-rheumatism drug; bDMARD: biologic disease modifying anti-rheumatism drug; MTX: methotrexate; NSAID: nonsteroidal anti-inflammatory drug; CRP: C-reactive protein; ESR: erythrocyte sedimentation rate; RF: rheumatoid factor; HAQ-DI: Health Assessment Questionnaire-Disability Index; AUC: area under the curve.

[a] Parameters were combined from 10 imputed datasets according to the Rubin's rule.

[b] LASSO models were estimated using two selected tuning parameter λ: λ_MAX to maximize the AUC, λ_1SE by the one-standard-error rule.

[c] These variables were transformed to resolve skewness before model development.

© 2023 Luo Y et al. *JAMA Network Open.*

eAppendix 1. Search Strategy

A. CENTRAL

#1 MeSH descriptor: [Certolizumab Pegol] explode all trees
#2 Certolizumab or “Certolizumab Pegol” or Cimzia or CDP870 or CDP-870 or “CDP 870” or “CTZ”
#3 (#1 or #2)
#4 MeSH descriptor: [Arthritis, Rheumatoid] explode all trees
#5 Rheumatoid arthritis
#6 RA
#7 (#4 or #5 or #6)
#8 #3 and #7

B. SCOPUS

KEY((certolizumab OR "certolizumab pegol" OR cimzia OR cdp-870 OR cdp870 OR "CDP 870" OR CTZ) AND ("rheumatoid arthritis" OR RA)) AND NOT INDEX (medline)

C. MEDLINE via Ovid (Medline(R))

1 exp certolizumab/
2 (certolizumab or ‘certolizumab pegol’ or cimzia or CDP870 or CDP-870 or ‘CDP 870’ or CTZ).mp.
3 or/1-2
4 exp Arthritis, Rheumatoid/
5 ("Rheumatoid Arthritis" or RA or (Caplan$ and Syndrome?) or (Felty$ and S?ndrome) or (Rheumatoid and Nodule?) or (Sjogren$ and S?ndrome?) or (Sicca$ and S?ndrome?) or (Ankylos$ and Spondylit$) or (Spondylarthritis and Ankylopoietica) or (Rheumatoid$ and Spondylit$) or (Bechterew$ and Disease?) or (Mari-Struempell and Disease?) or (Adult and Onset and Still$ and Disease?)).mp.
6 or/4-5
7 (3 and 6)
8 exp randomized controlled trials/
9 exp double-blind method/
10 exp single-blind method/
11 exp cross-over studies/
12 randomized controlled trial.pt.
13 clinical trial.pt.
14 controlled clinical trial.pt.
15 (random$ adj2 control$ adj2 trial$).mp.
16 (clinic$ adj2 trial$).mp.
17 random$.mp.
18 placebo$.tw.

19 assign$.tw.

20 allocat$.tw.

21 (crossover$ or cross over$ or cross-over$).mp.

22 factorial$.tw.

23 ((singl$ or double$ or trebl$ or tripl$) adj (blind$ or mask$)).mp.

24 or/8-23

25 (7 and 24)

© 2023 Luo Y et al. *JAMA Network Open.*

eAppendix 2. Details of the Statistical Analysis

2.1 IPD-MA: Average relative outcomes between two groups

Let y_{ij} be the outcome of interest ($y_{ij} = 1$ for the event, $y_{ij} = 0$ for non-event) for patient i where $i = 1, 2, \dots, n_j$ in trial j out of N trials; p_{ij} be the probability of the event; t_{ij} be the group of patient i in trial j was assigned to ($t_{ij} = 1$ for the intervention [CTZ + csDMARDs], $t_{ij} = 0$ for the control group [placebo + csDMARDs]):

$$y_{ij} \sim Bernoulli(p_{ij})$$

$$\log\left(\frac{p_{ij}}{1-p_{ij}}\right) = logit(p_{ij}) = \alpha_j + \delta_j t_{ij}$$

$$\delta_j \sim N(\delta, \tau^2)$$

Then, α_j is the log odds of the outcome for the control group in trial j, assumed independent across trials; δ_j is the treatment effect in terms of log OR in trial j, assumed exchangeable across trials; hence, δ is the summary estimate of the log-odds ratios for the intervention versus the control arm, and τ^2 is the heterogeneity of δ across trials. We used *R2Jags* package to fit this model[1].

2.2 Model: stage one

We fitted three penalized logistic regression models to estimate the probability of remission or low disease activity using baseline characteristics regardless of treatment (i.e., treatment was not included as a covariate in the model). Penalization methods to shrink the coefficients were used to avoid extreme estimations[2]. Before developing the model, we transformed the continuous variables which displayed noticeable skewness and checked the correlations between the continuous covariates (results are shown in eAppendix section 4. The models were:

(1) **LR22+PML**: Logistic regression of 22 covariates + penalized maximum likelihood (PML) shrinkage methods, where the tuning parameter was selected as the one that can maximize a modified Akaike's information criterion (AIC)[2 3]; the `lrm` and `pentrace` function from the *rms* package were used[4].

(2) **LR22+LASSO**: Logistic regression of 22 covariates + LASSO (least absolute shrinkage and selection operator) method, where two tuning parameter λ values were selected based on <u>10-fold cross validation</u> AUC: one to maximize the AUC, and another by the one-standard-error rule (i.e., the most parsimonious model whose AUC is no more than one standard error lower than that of the best model)[5 6]; the `glmnet` command from *glmnet* package was used[7].

(3) **LR16+PML**: Logistic regression of 16 *selected* covariates + PML, for which we conducted a literature review to select variables that have been reported to be important risk factors for disease activity in multiple previous studies[8]. The 16 selected variables and the references were provided in eAppendix section 3.

© 2023 Luo Y et al. *JAMA Network Open.*

The logistic regression part of all the models had the same form:

$$y_{ij} \sim Bernoulli(r_{ij})$$

$$logit(r_{ij}) = b_{0j} + \sum_{k=1}^{p} b_{kj} \times PF_{ijk}$$

r_{ij} is the probability of the outcome for patient i from trial j at baseline; PF_{ijk} is the kth prognostic factor in study j for patient i. Then, b_{0j} and b_{kj} are the intercept and the regression coefficient for the kth prognostic factor in study j; they are fixed across studies.

The outcome variable was measured repeatedly during the follow-up. Patients who had used rescue therapy before a follow-up visit were defined as non-responders at that visit, despite the measured disease activity scores. Then, we checked the missing pattern over time in the outcome variable for each study. We considered patients who had no outcome data at consecutive visits starting before three months until six months were unlikely to achieve response, thus we defined them as non-responders at the 3-month visit. Other than this situation, patients who had missing outcome data at the 3-month visit were treated as missing values. Finally, we used multiple imputation chained equation (`mice` function from the *mice* package[9]) to handle missing values in the covariates and outcomes for each model based on the missing-at-random assumption[10 11]. The variables used in the imputation model were the same as in the substantive model. Predictive mean matching was used for continuous variables, while logistic regression imputation for categorical variables. We imputed 10 datasets for each model and checked the convergence. We fitted the above three models on each imputed dataset, and combined the coefficients based on Rubin's rule.

We conducted bootstrap internal validation to estimate the optimism-corrected AUC, calibration intercept and slope for three stage-one models[2]. For each model, we first calculated the apparent performance in terms of the above metrics for the final model ($Model_{final}$) on the original dataset. Then, for each of the 10 imputed datasets M_i, we drew 200 bootstrap samples, and trained a model using the same modeling approach on each bootstrap sample ($Model_{bootstrap}$). Next, we calculated the apparent performance of $Model_{bootstrap}$ on the bootstrap sample, and the test performance of $Model_{bootstrap}$ on the imputed dataset M_i, and their difference was the optimism for $Model_{bootstrap}$. Then, we could get the average optimism for each M_i over 200 bootstrap samples, and the final optimism over 10 imputed datasets. The bootstrap optimism corrected AUC, calibration intercept and slope were computed by subtracting the final optimism from the apparent performance of $Model_{final}$. The bootstrap internal validation used self-programmed R-routines.

2.3 Model: stage two

$logit(r_{ij})$ is the logit of baseline expected probability of the outcome estimated at stage one,

and $\overline{logit(r_{ij})}$ is the average of logit-risk for all the individuals of the study population.

$$y_{ij} \sim Bernoulli(p_{ij})$$

$$logit(p_{ij}) = \begin{cases} a_j + g_{0j} \times \left(logit(r_{ij}) - \overline{logit(r_{ij})}\right), if\ t_{ij} = 0 \\ a_j + \delta_j + g_{0j} \times \left(logit(r_{ij}) - \overline{logit(r_{ij})}\right) + g_j \times \left(logit(r_{ij}) - \overline{logit(r_{ij})}\right), if\ t_{ij} = 1 \end{cases}$$

$$\delta_j \sim N(\delta, \tau^2)$$

$$g_{0j} \sim N(\gamma_0, \sigma_{\gamma_0}^2)$$

$$g_j \sim N(\gamma, \sigma_{\gamma}^2)$$

Then, a_j is the log odds a patient whose baseline expected probability of the outcome equals to the mean probability in the control group, and is assumed to be independent across trials; g_{0j} is the coefficient of the baseline expected probability, g_j is the coefficient of the effect modification of it, both assumed to be exchangeable cross trials and normally distributed about a summary estimate γ_0 and γ respectively.

This model was developed on patients with no missing values in all the baseline variables in the stage-one model (as the estimated baseline expected probability of the outcome from the stage-one model would be used) and the outcome variable. In one study (RAPID1[12]), all the 22 variables were measured, but the individual-level data for five variables (sex, BMI, duration, previous use of csDMARDs and biologics) was not provided. Hence, the baseline expected probabilities could not be estimated for all the patients from this trial at stage one. To solve it, we imputed the baseline expected probabilities by incorporating a Bayesian model, which took the reported aggregate-level means and standard deviations or proportions of these variables, to assume the prior distributions.

In order to estimate a new patient i who is not from any trials and has a logit baseline expected probability of the outcome $\widetilde{logit(r_i)}$ estimated from the stage-one model, we used the following equations:

$$logit(p_i) = a + \gamma_0 \times \left(\widetilde{logit(r_i)} - \overline{logit(r)}\right), if\ t_i = 0$$

$$logit(p_i) = a + \delta + \gamma_0 \times \left(\widetilde{logit(r_i)} - \overline{logit(r)}\right) + \gamma \times \left(\widetilde{logit(r_i)} - \overline{logit(r)}\right), if\ t_i = 1$$

δ, γ_0 and γ were estimated in stage-two, $\overline{logit(r)}$ was estimated as the mean of $logit(r_{ij})$ over all the individuals from all included studies; and a was estimated as the mean log-odds in all the control arms. Then we estimated the individual probability of the outcome if receiving CTZ+csDMARDs or placebo+csDMARDs respectively, and estimated the absolute risk difference between two groups. We used *R2Jags* package to fit this model[1].

© 2023 Luo Y et al. *JAMA Network Open.*

Note that this stage-two model could not be validated for its risk difference estimation like the stage-one model. It is because stage-two model aimed to estimate the risk difference of adding CTZ vs no CTZ. In order to evaluate whether the estimation is correct or not, we need the actual outcome of each patient. In this case, both the outcome when the patient received CTZ and the outcome when he/she did not receive CTZ were necessary. However, as a specific patient was treated by either CTZ or no CTZ, we could not acquire necessary data to assess the model performance. New methods have been proposed to evaluate the performance of models aiming to estimate personalized treatment effect between two groups very recently[13], which could be considered in future research.

eReferences

1. R2jags: Using R to Run 'JAGS' [program]. 0.7-1 version, 2021.
2. Steyerberg EW. Clinical Prediction Models: A Practical Approach to Development, Validation, and Updating. First. New York, N.Y.: Springer, New York, N.Y. 2009:500.
3. Moons KG, Donders AR, Steyerberg EW, et al. Penalized maximum likelihood estimation to directly adjust diagnostic and prognostic prediction models for overoptimism: a clinical example. *J Clin Epidemiol* 2004;57(12):1262-70. doi: 10.1016/j.jclinepi.2004.01.020
4. rms: Regression Modeling Strategies [program]. 6.2-0 version, 2021.
5. Hastie T, Tibshirani R, Jerome F. The Elements of Statistical Learning: Data Mining, Inference, and Prediction. Second. New York, N.Y.: Springer, New York, N.Y. 2009:745.
6. Tibshirani R. Regression Shrinkage and Selection Via the Lasso. *Journal of the Royal Statistical Society Series B (Methodological)* 1996;58(1):267-88.
7. Friedman J, Hastie T, Tibshirani R. Regularization Paths for Generalized Linear Models via Coordinate Descent. *Journal of Statistical Software* 2010;33(1):1-22. doi: 10.18637/jss.v033.i01
8. Steyerberg EW, Eijkemans MJ, Harrell FE, Jr., et al. Prognostic modelling with logistic regression analysis: a comparison of selection and estimation methods in small data sets. *Stat Med* 2000;19(8):1059-79. doi: 10.1002/(sici)1097-0258(20000430)19:8<1059::aid-sim412>3.0.co;2-0
9. van Buuren S, Groothuis-Oudshoorn K. mice: Multivariate Imputation by Chained Equations in R. *Journal of Statistical Software* 2011;45(3):1-67. doi: 10.18637/jss.v045.i03
10. Carpenter JR, Kenward MG. Missing data in randomised controlled trials: a practical guide. 2007.
11. White IR, Royston P, Wood AM. Multiple imputation using chained equations: Issues and guidance for practice. *Stat Med* 2011;30(4):377-99. doi: 10.1002/sim.4067
12. Keystone E, Heijde D, Mason D, Jr., et al. Certolizumab pegol plus methotrexate is significantly more effective than placebo plus methotrexate in active rheumatoid arthritis: findings of a fifty-two-week, phase III, multicenter, randomized, double-blind, placebo-controlled, parallel-group study. *Arthritis Rheum* 2008;58(11):3319-29. doi: 10.1002/art.23964
13. Efthimiou O, Hoogland J, Debray TPA, et al. Measuring the performance of prediction models to personalize treatment choice. *Stat Med* 2023;42(8):1188-206. doi: 10.1002/sim.9665

© 2023 Luo Y et al. *JAMA Network Open.*

eAppendix 3. Variable Selection Based on Previous Literature

We conducted a literature review to identify strong risk factors for low disease activity or remission. The selected 16 variables and references are listed in the table below.

<table>
<tr><th>Risk factors</th><th colspan="2">References</th></tr>
<tr><td>Age</td><td colspan="2">Xie-2019[1], Xiang-2020[2], Contreras-Yanez-2012[3]</td></tr>
<tr><td>Sex</td><td colspan="2">Capelusnik-2022[4], Koh-2021[5], Hamann-2019[6], Bird-2017[7], Jayakumar-2012[8], Xie-2019[1]</td></tr>
<tr><td>BMI</td><td colspan="2">Tipsing-2021[9], Lee-2021[10], Didden-2018[11], Hamann-2019[6], Bird-2017[7]</td></tr>
<tr><td>Disease duration</td><td colspan="2">Tipsing-2021[9], Capelusnik-2022[4], Didden-2018[11], Miwa-2017[12], Jayakumar-2012[8], Xie-2019[1], Xiang-2020[2]</td></tr>
<tr><td>Previous csDMARD use</td><td colspan="2">De Jong-2018[13], Didden-2018[11], Xie-2019[1], Kawashiri-2021[14]</td></tr>
<tr><td>Previous bDMARD use</td><td colspan="2">Didden-2018[11], Hamann-2019[6], Xie-2019[1]</td></tr>
<tr><td>Tender joint count</td><td>Miwa-2017[12], Jayakumar-2012[8], Hammer-2020[15]</td><td rowspan="7">*Disease activity composite scores:
De Jong-2018[13],
Yilmaz-Oner-2019[16],
Capelusnik-2022[4], Bird-2017[7],
de Punder-2015[17],
Xie-2019[1],
Xiang-2020[2]</td></tr>
<tr><td>Swollen joint count</td><td>Hamann-2019[6]</td></tr>
<tr><td>Patient global assessment</td><td>Hamann-2019[6], Kawashiri-2021[14]</td></tr>
<tr><td>Physician global assessment</td><td>Miwa-2017[12], Kawashiri-2021[14]</td></tr>
<tr><td>Patient assessment of pain</td><td>De Jong-2018[13], Yilmaz-Oner-2019[16], Capelusnik-2022[4]</td></tr>
<tr><td>CRP</td><td>Ma-2020[18], de Punder-2015[17], Contreras-Yanez-2012[3]</td></tr>
<tr><td>ESR</td><td>Hamann-2019[6], Miwa-2017[12]</td></tr>
<tr><td>Rheumatoid factor</td><td colspan="2">Didden-2018[11], Kawashiri-2021[14]</td></tr>
<tr><td>HAQ-DI</td><td colspan="2">Lee-2017[19], Yilmaz-Oner-2019[16], Hamann-2019[6]</td></tr>
<tr><td>Patient assessment of fatigue</td><td colspan="2">Seifert-2019[20], Doumen-2021[21]</td></tr>
</table>

Abbreviation: BMI: body mass index; csDMARD: conventional synthetic disease modifying anti-rheumatism drug; bDMARD: biologic disease modifying anti-rheumatism drug; HAQ-DI: Health Assessment Questionnaire-Disability Index.

(1) We found two other critical risk factors reported in multiple previous literature: smoking[5 6 12], anti-CCP[13 16 17]. Since they were not provided in some studies, we could not include them in our model.

(2) Tender/swollen joint count, patient/physician's global assessment, patient's assessment of pain, ESR/CRP are constituents of composite disease activity scores. In some studies, baseline disease activity score as a whole was suggested as a strong risk factor for low disease activity/remission.

eReferences

1. Xie W, Li J, Zhang X, et al. Sustained clinical remission of rheumatoid arthritis and its predictive factors in an unselected adult Chinese population from 2009 to 2018. *Int J Rheum Dis* 2019;22(9):1670-78. doi: 10.1111/1756-185X.13651

2. Xiang Y, Wang Q, Li H, et al. Chinese registry of rheumatoid arthritis (CREDIT): III. The transition of disease activity during follow-ups and predictors of achieving treatment target. *Int J Rheum Dis* 2020;23(12):1719-27. doi: 10.1111/1756-185X.13996

3. Contreras-Yanez I, Rull-Gabayet M, Pascual-Ramos V. Early disease activity suppression and younger age predict excellent outcome of recent-onset rheumatoid arthritis patients treated with conventional disease modifying anti-rheumatic drugs. *Clin Exp Rheumatol* 2012;30(3):402-8.

4. Capelusnik D, Aletaha D. Baseline predictors of different types of treatment success in rheumatoid arthritis. *Ann Rheum*

© 2023 Luo Y et al. *JAMA Network Open.*

Dis 2022;81(2):153-58. doi: 10.1136/annrheumdis-2021-220853
5. Koh JH, Lee SK, Kim J, et al. Effectiveness and safety of biologic and targeted synthetic disease-modifying anti-rheumatic drugs in elderly patients with rheumatoid arthritis: real-world data from the KOBIO Registry. *Clin Exp Rheumatol* 2021;39(2):269-78.
6. Hamann PDH, Pauling JD, McHugh N, et al. Predictors, demographics and frequency of sustained remission and low disease activity in anti-tumour necrosis factor-treated rheumatoid arthritis patients. *Rheumatology (Oxford)* 2019;58(12):2162-69. doi: 10.1093/rheumatology/kez188
7. Bird P, Nicholls D, Barrett R, et al. Longitudinal study of clinical prognostic factors in patients with early rheumatoid arthritis: the PREDICT study. *Int J Rheum Dis* 2017;20(4):460-68. doi: 10.1111/1756-185X.13036
8. Jayakumar K, Norton S, Dixey J, et al. Sustained clinical remission in rheumatoid arthritis: prevalence and prognostic factors in an inception cohort of patients treated with conventional DMARDS. *Rheumatology (Oxford)* 2012;51(1):169-75. doi: 10.1093/rheumatology/ker250
9. Tipsing W, Sawanyawisuth K. Predictive clinical factors in rheumatoid arthritis using disease activity and functional score. *Reumatologia* 2021;59(5):309-12. doi: 10.5114/reum.2021.110611
10. Lee SY, Ibrahim F, Tom BDM, et al. Baseline predictors of remission, pain and fatigue in rheumatoid arthritis: the TITRATE trial. *Arthritis Res Ther* 2021;23(1):278. doi: 10.1186/s13075-021-02653-1
11. Didden EM, Ruffieux Y, Hummel N, et al. Prediction of Real-World Drug Effectiveness Prelaunch: Case Study in Rheumatoid Arthritis. *Med Decis Making* 2018;38(6):719-29. doi: 10.1177/0272989X18775975
12. Miwa Y, Saito M, Furuya H, et al. Predictor of the Simplified Disease Activity Index 50 (SDAI 50) at Month 3 of bDMARD Treatment in Patients with Long-Established Rheumatoid Arthritis. *Open Rheumatol J* 2017;11:106-12. doi: 10.2174/1874312901711010106
13. de Jong TD, Sellam J, Agca R, et al. A multi-parameter response prediction model for rituximab in rheumatoid arthritis. *Joint Bone Spine* 2018;85(2):219-26. doi: 10.1016/j.jbspin.2017.02.015
14. Kawashiri SY, Endo Y, Okamoto M, et al. Contributing factors of clinical outcomes at 1 year post-diagnosis in early rheumatoid arthritis patients with tightly controlled disease activity in clinical practice: a retrospective study. *Mod Rheumatol* 2021;31(2):343-49. doi: 10.1080/14397595.2020.1795392
15. Hammer HB, Michelsen B, Provan SA, et al. Tender Joint Count and Inflammatory Activity in Patients With Established Rheumatoid Arthritis: Results From a Longitudinal Study. *Arthritis Care Res (Hoboken)* 2020;72(1):27-35. doi: 10.1002/acr.23815
16. Yilmaz-Oner S, Gazel U, Can M, et al. Predictors and the optimal duration of sustained remission in rheumatoid arthritis. *Clin Rheumatol* 2019;38(11):3033-39. doi: 10.1007/s10067-019-04654-7
17. de Punder YM, Jansen TL, van Ede AE, et al. Personalizing treatment targets in rheumatoid arthritis by using a simple prediction model. *J Rheumatol* 2015;42(3):398-404. doi: 10.3899/jrheum.140085
18. Ma MHY, Defranoux N, Li W, et al. A multi-biomarker disease activity score can predict sustained remission in rheumatoid arthritis. *Arthritis Res Ther* 2020;22(1):158. doi: 10.1186/s13075-020-02240-w
19. Lee KE, Choi SE, Xu H, et al. HAQ score is an independent predictor of sustained remission in patients with rheumatoid arthritis. *Rheumatol Int* 2017;37(12):2027-34. doi: 10.1007/s00296-017-3833-z
20. Seifert O, Baerwald C. Impact of fatigue on rheumatic diseases. *Best Pract Res Clin Rheumatol* 2019;33(3):101435. doi: 10.1016/j.berh.2019.101435
21. Doumen M, De Cock D, Pazmino S, et al. Psychosocial burden predicts sustained remission in early rheumatoid arthritis: unraveling the complex interplay of wellbeing and disease activity. *Arthritis Care Res (Hoboken)* 2021 doi: 10.1002/acr.24847

© 2023 Luo Y et al. *JAMA Network Open.*

eAppendix 4. Variable Preprocessing

4.1 Transformation of five continuous variables

(1) Duration from onset (years):

\# Before transformation:

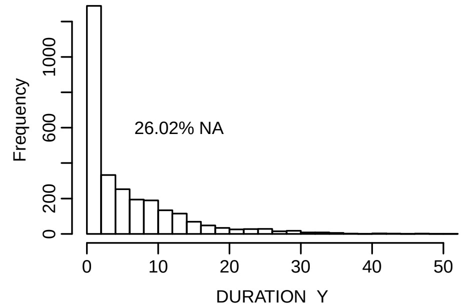

\# After transformation: *log transformation*

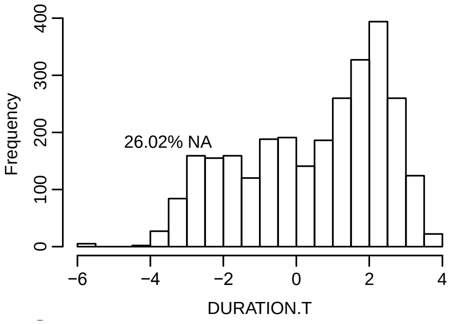

(2) Age (years): *Note: Only age band was provided for each patient in the dataset, and the cut-offs were different from study to study. Therefore, we took the median age in the band for each patient.

\# Before transformation:

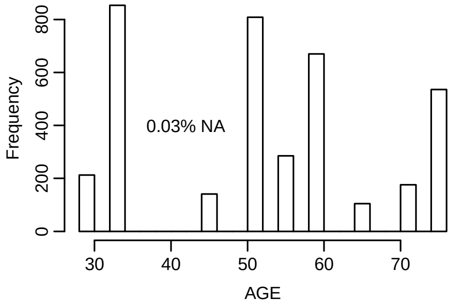

\# After transformation: *log transformation*

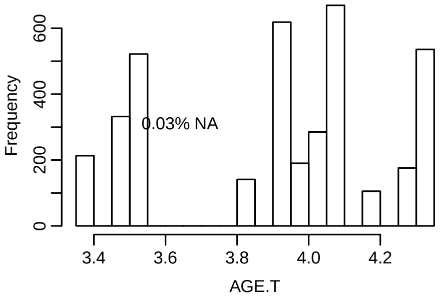

(3) Tender joint count (TJC):

\# Before transformation:

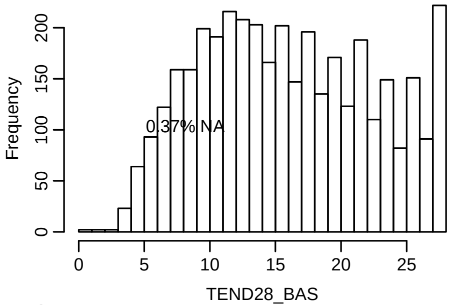

\# After transformation: *(TJC+1)^0.6*

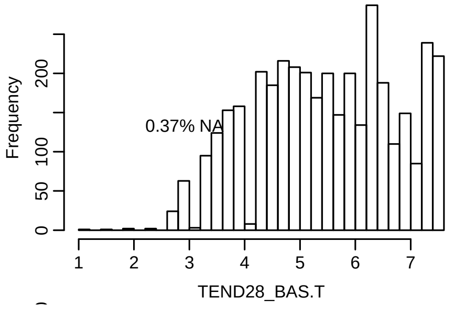

© 2023 Luo Y et al. *JAMA Network Open.*

(4) CRP (mg/L):

Before transformation:

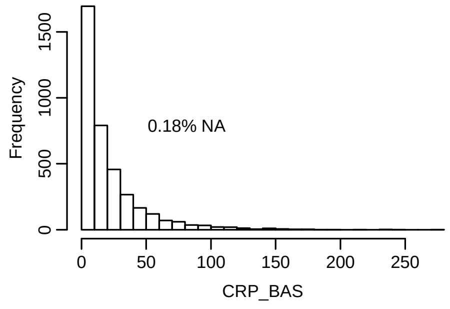

After transformation: *log(CRP+1)*

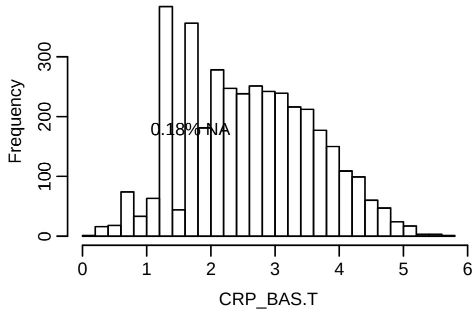

(5) RF (IU/mL):

Before transformation:

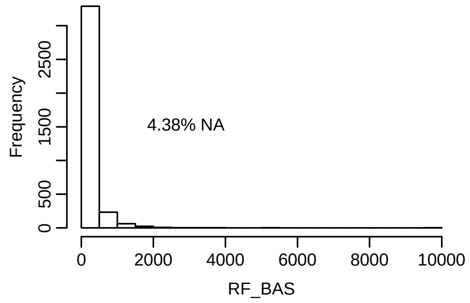

After transformation: 1/*(RF+1)^0.1)*

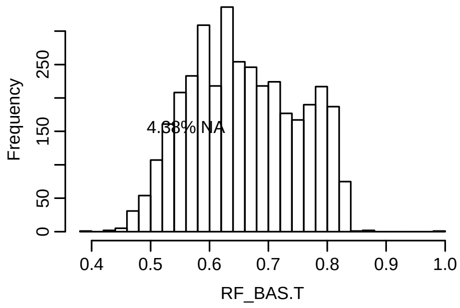

4.2 Correlation coefficients between continuous variables

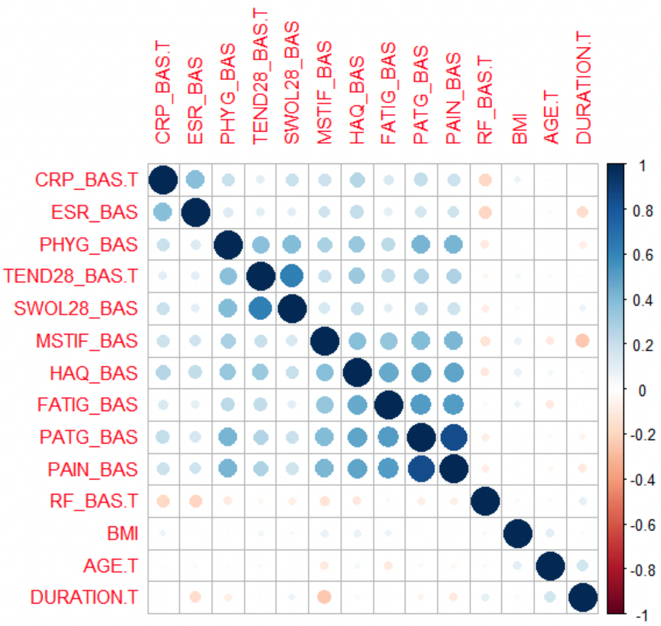

CRP_BAS.T	CRP
ESR_BAS	ESR
PHYG_BAS	Physician's global assessment
TEND28_BAS.T	Tender joint count (0-28)
SWOL28_BAS	Swollen joint count (0-28)
MSTIF_BAS	Morning stiffness
HAQ_BAS	HAQ-DI score
FATIG_BAS	Fatigue score
PATG_BAS	Patient's global assessment
PAIN_BAS	Patient's assessment of pain
RF_BAS.T	RF
BMI	BMI
AGE.T	Age
DURATION.T	Disease duration

There are two pairs whose correlation coefficients are higher than 0.6: patient's global assessment and patient's assessment of pain: 0.85; tender joint count and swollen joint count: 0.62. Considering these two variables were both critical items in most disease activity indices, and models using the PML method did not perform variable selection, we kept both variables in all the models.

© 2023 Luo Y et al. *JAMA Network Open.*
