# Supplementary material for: Estimating Patient-Specific Relative Benefit of Adding Biologics to Conventional Rheumatoid Arthritis Treatment: An Individual Participant Data Meta-Analysis
Source: JAMA Netw Open. 2023 Jun 30;6(6):e2321398. doi: 10.1001/jamanetworkopen.2023.21398 (PMC10314313; doi:10.1001/jamanetworkopen.2023.21398)
Supplement: Supplement 2. — Data Sharing Statement [file jamanetwopen-e2321398-s002.pdf]

# Data Sharing Statement

Luo. Estimating Patient-Specific Relative Benefit of Adding Biologics to Conventional Rheumatoid Arthritis Treatment. *JAMA Netw Open*. Published June 30, 2023. doi:10.1001/jamanetworkopen.2023.21398

**Data**
**Data available:** No

**Additional Information**
**Explanation for why data not available:** The data that support the findings of this study are available from http://vivli.org but restrictions apply to the availability of these data, which were used under license for the current study, and so are not publicly available. Data are however available from http://vivli.org upon reasonable request and application, after their permission.
